# Fournier's gangrene: Seven years of experience in the emergencies service of visceral surgery at Ibn Rochd University Hospital Center

**DOI:** 10.1016/j.amsu.2021.102821

**Published:** 2021-10-30

**Authors:** F.Z. Bensardi, A. Hajri, Sylvestre Kabura, M. Bouali, A. El Bakouri, K. El Hattabi, A. Fadil

**Affiliations:** a Service of Emergency of Visceral Surgery, Ibn Rochd-Casablanca University Hospital Centre, Morocco; b Hassan II University of Casablanca, Medicine and Pharmacy Faculty, Morocco; c Department of Surgery, Ibn Rochd-Casablanca University Hospital Centre, Morocco; d Service of Digestive Cancer Surgery and Liver Transplantation, Morocco

**Keywords:** Fournier's gangrene, Cleanliness stoma, Antibiotic therapy, Necrosectomy

## Abstract

**Introduction:**

This work aims to describe and discuss the epidemiological, clinical, therapeutic and evolution of Fournier's gangrene.

**Materials and methods:**

Case series with retrospective data collection of patients treated for Fournier's gangrene between January 2010 and March 2017. The main etiologies, risk factors, postoperative complications outcomes and long term follow up results were analyzed.

**Results:**

Eight four (84) patients were recruited. The average age of our patients was 49 years (with limits of 20–76), the male gender dominates our series (83.33%) with a sex ratio of 5 M/1W, the most frequently found risk factor was diabetes mellitus (37%). The most common etiology was anal abscesses (32%). The average time to consultation was 8 days (limits ranges from 3 to 30 days). All patients were admitted at a necrosis stage (100%). Anemia was identified in 85% of cases. The low platelets were noticed in 44.03% of cases. Hypoalbuminemia was found in 93% of cases. All patients (100%) benefited resuscitation initially and antibiotic therapy on their admission. They received emergency surgical debridement with a cleansing stoma. The average length of hospital stay was 13 days and complications occurred in 33% of cases. The mortality rate was 7.14%.

**Conclusion:**

Fournier's gangrene is a medico-surgical emergency with a high morbidity and mortality rate. Early diagnosis as well as antibiotic therapy and the quality of debridement save the patients.

## Highlights

• Fournier's gangrene is a rare but severe disease.
• The diagnosis and treatment delay and lesions extension increase mortality.
• The treatment is multidisciplinary.
• Early diagnosis and treatment improve prognosis.
• Tobacco intoxication is suspected to be risk factor.

## 1 Introduction

Fournier's gangrene is a necrotizing fasciitis of the soft tissues of the perineum, external genitalia area and the perianal region of infectious origin by aerobic and/or anaerobic bacteria with a synergistic action [1]. The etiology is identified in 95% of cases. Risk factors are often present in patients with this pathology [2]. The diagnosis is essentially clinical and imaging assessments are for differential diagnosis and evaluation of the complications [3,4]. Despite the progress in techniques and methods of anesthesia and resuscitation, mortality remains high, at around 20–30%. The therapeutic management is medico-surgical [5,6]. The aim of our study is to report our experience on perineal gangrene and review its epidemiology, risk factors, etiologies, diagnostic means, and therapeutic strategies. This manuscript is presented in line with process cheicklist 2020 Criteria [7].

## 2 Materials and methods

This is a retrospective study during 7 years and 3 months from January 1, 2010 to March 31, 2017 involving 84 patients with Fournier's gangrene hospitalized in the service of visceral surgery emergencies P35 of the IBN ROCHD university hospital center of Casablanca with infectious or idiopathic origin. Confirmed cases with an operative report were included. Anal abscesses, cellulitis and patients with incomplete files were excluded from our study. The data was collected with data collection sheets from the archives of patient's files. Statistical analysis was performed using the epi-info 7.0 version. The qualitative values were expressed as percentage while the quantitative values were expressed as mean and standard deviation and were checked for normality.

## 3 Results

The average age of our patients was 49 years (with limits of 20–76 years), the male sex represented 83.33% and the sex ratio was 5 M/1W. The most common risk factor was diabetes (37% of cases) and the anal abscess was the most common etiology (32%). The consultation time was 8 days with ranges from 3 to 30 days. The Glasgow coma scale was altered on admission in 15 patients. The risk factors found were mainly diabetes, followed by tobacco and alcoholism (Table 1). The etiology of Fournier's gangrene was found in 57 cases, or 68%. Thus, 32% of cases had developed Fournier's gangrene complicating anal abscess; 11 patients (13.1%) had a hemorrhoidal pathology; nine patients (11%) presented with anal fissure, seven patients (8.3%) had anal fistula. A patient was followed for rectal adenocarcinoma for which he received several sessions of radiotherapy and chemotherapy; two patients had developed the disease following perineal trauma. In 27 patients (32%), no etiology was found (Table 2).

**Table 1:** Risk factors founded in our study.

Past medical history and comorbidities	Number of cases	Percentage %
Diabetes	31	37
- Type 2	28	
- Type 1	3	
Tobacco	40	48
Cardiovascular pathologies	6	7.10
Malignant hemopathy	1	1.19
Rectal adenocarcinoma	1	1.19
Retroviral infections	1	1.19
Nodes Tuberculosis	1	1.19
Prolonged corticosteroids therapy	1	1.19
Morbid obesity	3	3.57
Alcohool	11	13.09
Paraplegia	1	1.19
None	27	32.10

**Table 2:** The main etiologies found in the patients of our study.

Etiology	Number of cases	Percentage %
Anal abcesses	27	32
Hemorrhoidal pathology	11	13.1
Anal Fissure	9	11
Anal fistula	7	8.3
Carcinome rectal	1	1.2
Traumatic	2	2.4
None	27	32
TOTAL	84	100

**Fig. 1:**
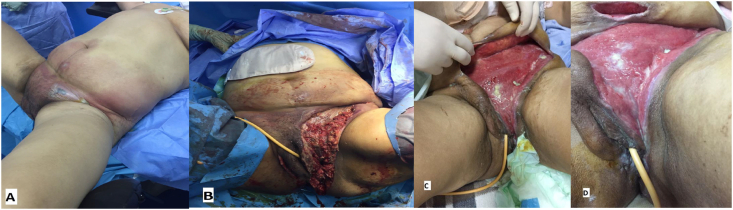
FG extended to inguinal regions and abdominal wall (A); note the epigastric location of the transverse diversion colostomy (B); C&D are the evolution of the patient after necrosectomy and antibiotic therapy with wound dressing.

Perineal pain was found in 94% of cases. Fever was present in 54 patients (64.26%) on admission. Fifty patients (59.5%) presented with tachycardia. Thirty-one patients (31) or 36.89% of cases were hypotensive on admission. The physical examination found inflammation with erythema and edema of the perineum in 100% (Fig. 1), Necrosis in 100%, posterior perineum in 50% of cases, Anterior perineum in 46% of cases and abdominal wall in 4% of cases (Table 3, Table 4). Anemia was identified in 85% of cases with hyperleukocytosis (89%) and hyperplaquetosis (30%). The low platelets were found in 44.03% of cases. Hyponatremia was the most common fluid electrolyte disorder on admission (44%) and hypernatremia in 2% of cases. Hypoalbuminemia was found in 93% of cases. Hyperkalaemia was reported in 17% of cases; and Hypokalaemia in 10% of cases. Acute functional renal failure on admission was noted in 31 patients (36.89%) of cases with normal function retake in 100% after rehydration. Capillary test of glycemia noted hyperglycemia in 46.41% of cases, while the fasting control sample revealed only 37% of hyperglycemia on admission. Hypoalbuminemia was found in 93% of cases. Hypoprotidemia was found in 60% of cases. The aminoaspartate transferase rate was high in 32% of cases and alanine aminotranspeptidase was elevated in 14% of cases.

**Table 3:** Physical signs found in our patients.

Physical signs	Number of cases	Percentage %
Perineal infiltration, erythema and necrosis	84	100
Perianal abcesses	27	32
fluctuate appearance of the perineum	24	29
Discharge of pus	33	40
Anal Fissure	9	11
Anal fistula	7	8.33
Snowy crackle	9	11

**Table 4:** Location and extension of necrosis of the patients of our study.

Location and extension of necrosis	Number of cases	Percentage %
Posterior perineum necrosis	42	50
Anterior perineum necrosis:	39	46.43
- Perineum and scrotal	31	
- Perineum and scrotal with penis extension	8	
Abdominal wall necrosis:	3	3.57
- Hypogastric	2	
- Lumbar	1	
Total	84	100

**Fig. 2:**
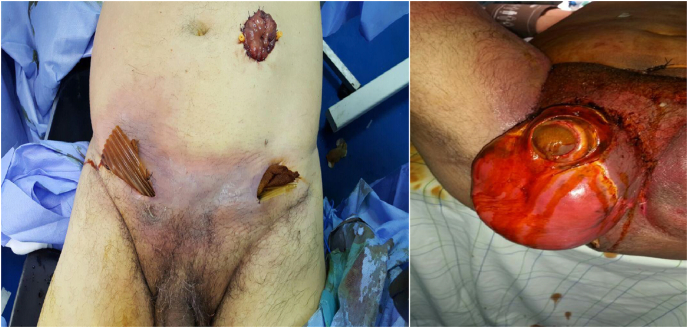
Patient with FG extended to the abdomen: Sigmoid diversion cleanliness colostomy in the left with cystostomy in the right.

**Fig. 3:**
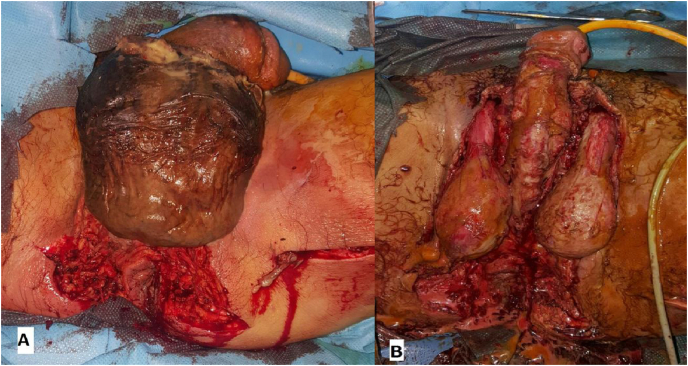
A: perineal surgical debridement in a patient of our study with FG extended to the anterior perineum and scrotum; B: The same patient after complete debridement.

All patients were treated by resuscitation measures and antibiotic therapy combining β-lactams, aminoglycosides and imidazoles on admission (57.16%). They had been operated in emergency with a stoma diversion in 100%; sigmoid in 85.72% of cases, transverse colon in 11.9% of cases and ileal in the rest of cases due to extension of the necrosis at abdominal wall and difficulties of exteriorization of sigmoid and transverse colon. (Fig. 2). Necrosectomy was limited to the posterior perineum in 50% of cases, extended to the anterior perineum (46%), abdominal wall (3.57%). Drainage was performed in 49% of cases, with 93% by Delbet drain (Fig. 3).

The dressing was done once a day in the operating room for the first days, either under sedation in 10.71% of cases or without sedation, in 89.25% of cases. The frequency of dressings had been reduced based on the improvement in the local condition. Washing of the wound was done with saline combined with dilute hydrogen peroxide and Polyvidone iodine. The dressing was made with betadinated or silver sulfadiazine (FLAMMAZINE®) dressings in the cleansing phase. During the budding phase, the pro-inflammatory dressings were made based on fat (Vaseline dressings). The patients who presented hypertrophic budding benefited from anti-inflammatory dressings based on topical corticosteroids (Cleniderm® or Diprolene®).

The average length of hospital stay was 13 days. Complications occurred in 33% of cases. Six patients had died during their hospitalization, for a mortality rate of 7.14%. The mean age of the deceased patients was 52.8 years, the sex was male and had consulted with a delay of more than 4 days and a mean time to diagnosis of 14.6 days. Three patients (50%) were diabetics and three others (50%) presented with gangrene extended beyond the perineum with 2 cases of abdominal extension and one case of extension to the sacral region). The cause of death was sepsis in 66% of cases, decompensation of other pathologies in 34% of cases (including 2 following diabetic ketoacidosis).

**Fig. 4:**
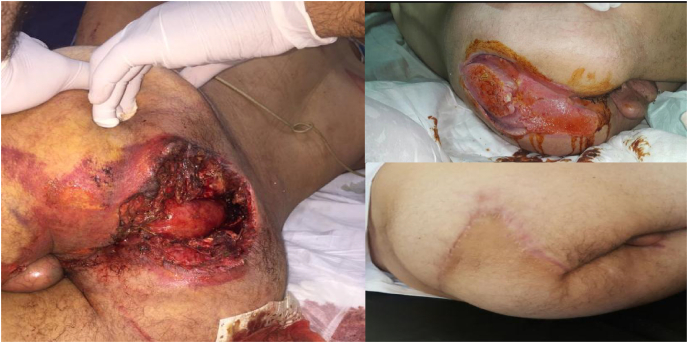
Traumatic FG: initial lesions on admission (A), evolution under expectative cicatrisation with wound dressing (B&C) and final aspect after complete healing and reconstructive surgery with muscle and cutaneous flap (D&E).

The stoma reversion was achieved in 70 patients (89.7% out of 78) within 6 months, with limits of 3–16 months depending on the speed of healing and the need for plastic surgery (Fig. 4). Eight patients (9.5%) had been lost to follow-up.

## 4 Discussion

Fournier's gangrene was first described in 1833 by Professor Jean-Alfred Fournier, a French dermatologist venerologist. Two important series have been published with great differences in the epidemiological data, a retrospective review from 1950 to 1999, by EKE had identified 1726 cases [8], and that by Sorensen et al., on 1641 patients [9]. The incidence of FG is 1.6 cases per 100,000 men per year which represented 0.02% of all hospital admissions [10]. The mean age of the patients was 50.9 years with a sex ratio of 10/1 M/F for the same series. Chen et al. [11] found an average age of 57.2 years within limits of 20–89 years. For our study, we found an average age of 49 years with the limits of 20–76 years. The sex ratio was 5 male/1 female and this is in accordance with other studies. The most common risk factor is diabetes in 37% of cases, followed by alcoholism in 13% of cases and cardiovascular pathologies in 7% of cases. Dekou [12] found diabetes as a risk factor in 14% while Ettalbi [13] found it in 35%. While in the literature, the authors do not insist on smoking as a risk factor for Fournier's gangrene, it was found in 48% of cases in our patients. However, it remains to be proven whether tobacco would be among the risk factors for the occurrence of gangrene of the perineum.

The diagnosis is essentially clinical. The physical signs can be local at the initial stage or general at advanced stage, which can lead to septic shock. There are many differential diagnoses including strangulated hernia in the phlegmon stage, scrotal or ischiorectal abscess, balanitis, herpes infection, pyoderma gangrenosum, polyarteritis nodosa, warfarin necrosis and ecthyma gangrenosum [14]. The bacterial strains responsible for FG consist of aerobic and anaerobic Gram-negative and positive species, with less frequency for anaerobic and snowy crackles manifestation on clinical examination. The principle found bacteria are: *E. coli*, *P. aeruginosa*, Proteus, Klebsiella, Streptococcus species, *S. aureus*, Enterococcus, Clostridia, bacteroides and less commonly multiresistant staphylococcus aureus and Candida in longer hospitalized patients [15].

Imaging assessment can help to differentiate the diagnosis of gangrene from other diseases, as scrotal edema and simple cellulitis. The use of bedside ultrasound is a quick and easy diagnostic tool that helps establish differential diagnoses [1]. Imaging is also necessary to confirm the diagnosis in case of doubt, to investigate the etiology and to assess the extent of the disease in cases of very advanced gangrene. Standard X-ray shows gas within soft tissue with a sensitivity of 90–100% before the clinically found snowy sign which sensitivity is 19–64% [3,16].

The medical and surgical management is based on emergency resuscitation of the patient, broad-spectrum antibiotic therapy and surgical debridement. The goal of treatment is to reduce systemic toxicity, stop the progression of the infection, and eliminate the germs involved. Early and radical excision of necrotic and devitalized tissue is the crucial step for stopping the progression of the infection. In a retrospective analysis of 72 patients with Fournier's gangrene, Yan et al. [17] noticed that a delay in treatment was associated with significant mortality [10]. In our study, all patients had undergone necrosectomy and a cleanliness stoma; sigmoid in 85.72% of cases, transverse in 11.9% of cases and ileal for other cases due to abdominal extension and difficulties with exteriorization of the sigmoid and transverse colon. The surgical debridement was extended to the posterior and anterior perineum and even to the inguinal regions depending on the extent of the necrosis and was most often repeated as necessary with dressings under general anesthesia. When the infection was controlled and the tissues were healthy, reconstructive plastic surgery was performed if necessary [10] [18]. In the literature, the combination of other methods of treatment including hyperbaric oxygen therapy and the application of negative pressure are used [19]. Some cases with extensive urethral or penis involvement, suprapubic cystostomy for urinary diversion may be indicated, but generally a urethral catheter provides a satisfactory diversion. Colostomy is reserved only for some patients with anorectal and sphincter area involvement at high risk of fecal contamination. Colostomy could be performed for patients who have sphincter lesions or those requiring extensive perianal debridement. In the literature, there are studies suggesting that the use of intestinal catheters (Bowel management catheter: BMC) or stool management Kit(SMK) to prevent fecal contamination may be beneficial to patients with severe lesions of the perianal area other than the GF [20,21]. Eray et al. applied this method for Fournier's gangrene and found satisfactory results and positive effects of the methods compared to patients who had stool diversion in terms of length of hospital stay and cost. In our department, we use cleansing colostomies to allow rapid healing and prevent contamination of debridement wounds by stool. In his article on the indications of stomy, patients with FG of proctologic origin require more cleanliness stomas compared to those of urologic origin [22]. All patients had as etiology proctologic origin. Despite well-adapted medical and surgical treatment and adequate resuscitation, mortality in FG is for Eke [8] 16% in his series of 1728 patients. Sorensen et al. reported 7.5% mortality for males and 12.8% for females, but these differences were not statistically significant. For our study, we found a death rate of 7.14%, which is comparable to data from other series.

## 5 Conclusion

Fournier's gangrene is a rare but severe disease. It is a high medico-surgical emergency. Its management is multidisciplinary, combining aggressive surgical treatment, broad-spectrum antibiotic therapy, then adapted, and intensive resuscitation. Its morbidity and mortality rate are high. Diagnosis and therapeutic time, extension of the lesions and the quality of debridement are factors of prognosis. Reconstructive surgery is often necessary.

## Disclosures

Provenance and peer review: Not commissioned, externally peer-reviewed.

## Conflicts of interest

No conflicts of interest.

## Sources of funding

No funding for research.

## Ethical approval

The study is exempt from ethical approval in our institution.

## Consent

Written informed consent was obtained from the patient for publication of this case.

## Author contribution

Fatima Zahra Bensardi: designed the study, wrote the protocol and the first draft of the manuscript. Hajri AMAL: designed the study, wrote the protocol and the first draft of the manuscript. KABURA Sylvestre: designed the study, wrote the protocol and the first draft of the manuscript. Mounir Bouali: managed the analyses, and the correction of the manuscript. Khalid ElHattabi: managed the analyses, and the correction of the manuscript. ElBakouri Abdelillah: managed the analyses, and the correction of the manuscript. Fadil Abdelaziz: managed the analyses, and the correction of the manuscript. All authors read and approved the final manuscript.

## Registration of research studies

Name of the registry: ClinicalTrials.gov.

2. Unique Identifying number or registration ID: NCT 04983056.

3. Hyperlink to the registration (must be publicly accessible):

https://clinicaltrials.gov/ct2/show/NCT04983056

## Guarantor

KABURA Sylvestre.
